# Building a predictive model for assessing the risk of *Salmonella* shedding at slaughter in fattening pigs

**DOI:** 10.3389/fmicb.2023.1232490

**Published:** 2023-08-23

**Authors:** María Bernad-Roche, Clara María Marín-Alcalá, Alberto Cebollada-Solanas, Ignacio de Blas, Raúl Carlos Mainar-Jaime

**Affiliations:** ^1^Departamento de Patología Animal, Facultad de Veterinaria, Instituto Agroalimentario de Aragón-IA2, Universidad de Zaragoza-CITA, Zaragoza, Spain; ^2^Departamento de Ciencia Animal, Centro de Investigación y Tecnología Agroalimentaria de Aragón, Instituto Agroalimentario de Aragón-IA2, Universidad de Zaragoza-CITA, Zaragoza, Spain; ^3^Unidad de Biocomputación, Instituto Aragonés de Ciencias de la Salud (IACS/IIS Aragón), Centro de Investigación Biomédica de Aragón (CIBA), Zaragoza, Spain

**Keywords:** prediction model, *salmonella* control, abattoir, shedding, swine

## Abstract

Salmonellosis continues to be a major cause of foodborne outbreaks worldwide, and pigs are one of the main sources of human infection. *Salmonella* pork contamination is a major concern for abattoirs and is related to the presence of *Salmonella* in pigs' feces at slaughter. Being able to predict the risk of *Salmonella* shedding in pigs arriving at the slaughterhouse could help mitigate abattoir and carcass contamination. For this purpose, 30 batches of 50 pigs each were selected from 30 different fattening units. The pigs were tagged and bled for the detection of antibodies against *Salmonella* approximately one month before slaughter. Pooled floor fecal samples were also collected from 10 pens per unit for *Salmonella* detection, and a questionnaire on biosecurity was administered to each farm. At the abattoir, colon content was collected from each tagged pig for the *Salmonella* shedding assessment. A predictive model for *Salmonella* shedding at slaughter was built with two-third of the pigs by employing random-effects logistic regression analysis, with *Salmonella* shedding as the dependent variable and pig serology and other farm/environmental characteristics as the independent variables. The model included farm as the grouping factor. Data from the remaining one-third of the pigs were used for model validation. Out of 1,500 pigs initially selected, 1,341 were identified at the abattoir and analyzed. *Salmonella* was detected in 13 (43.3%; 95%CI = 27.4–60.8) of the fattening units. The mean batch seroprevalence (cut-off OD% ≥40) among the fattening units was 31.7% (95%CI = 21.8–41.0), and a total of 316 pigs (23.6%; 95%CI = 21.4–25.9) shed *Salmonella* at slaughter. The model predicted reasonably well (Area under the curve = 0.76; *P* < 0.05) whether a pig would shed *Salmonella* at slaughter, with estimates of sensitivity and specificity at 71.6% and 73.6%, respectively. Serology, the percentage of *Salmonella*-positive pens on the farm, and the internal biosecurity score were significantly associated (*P* < 0.05) with *Salmonella* shedding at the abattoir, and several scenarios were observed by the model. The study highlighted that although serology may be helpful for identifying batches of pigs at risk of shedding *Salmonella* upon their arrival at the abattoir, it may not be necessary in some scenarios.

## 1. Introduction

Salmonellosis remains one of the most frequent foodborne zoonoses in the EU, with 60,050 human cases (15.7/100,000 inhabitants) in 2021. In the last year, *S*. Enteritidis, *S*. Typhimurium, and the monophasic variant of *S*. Typhimurium (mST) were among the most reported serovars, with the latter two mainly associated with contaminated pork (EFSA and ECDC, 2022).

In contrast to the fowl industry, to date, few EU countries have established National Control Programs (NCPs) against pig salmonellosis. On-farm *Salmonella* control programs in fattening swine were expected to be initiated in all EU after Regulation EC No. 2160/2003. However, most EU countries did not implement them, likely because they were not considered cost-effective (Anonymous, 2011).

The first comprehensive *Salmonella* NCP for pigs in Europe was established in Sweden in the 60s (Wierup, 2006) after a major food-borne outbreak in 1953 (Lundbeck et al., 1955), which was followed later by Norway, Finland, and Denmark in 1995 (Mousing et al., 1997; Maijala et al., 2005; Lyngstad et al., 2007). All but Denmark's were focused on eradication and had bacteriological analyses as the keystone. Denmark developed its own NCP based on both bacteriological and serological analyses, and its focus was mainly on *Salmonella* control. In all cases, the farm-level prevalence was initially low, and strict measures were enforced when *Salmonella* was found. These measures included the application of economic penalties. Positive results were observed in reducing the overall *Salmonella* prevalence in pig carcasses but at a high cost (Anonymous, 2011).

After the success of the Scandinavian action plans and along with EU regulation, new NCPs followed suit in other European countries: Germany and United Kingdom in 2002 (Osterkorn et al., 2001; BPEX, 2002; Snary et al., 2010), Ireland in 2003 (Statutory Instrument No. 165/2002), the Netherlands in 2005 (Hanssen et al., 2007), and Belgium in 2007 (Méroc et al., 2012). In general, these programs were similar to the Danish NCP, focusing on control, but they were based mostly on serological analysis of a relatively small number of pigs per batch slaughtered. Thus, pig herds were categorized into three different risk groups: low-risk (I), medium-risk (II), and high-risk herds (III). Category III herds had to undertake farm-specific activities aimed at reducing their *Salmonella* exposure and, accordingly, their *Salmonella* seroprevalence. Although no penalties were generally applied, incentives were offered to farmers in some countries, such as to be included in pork quality assurance schemes, particularly, the Qualität und Sicherheit (QS) in Germany, the British Quality Assured Pork (BQAP) in the UK, the Bord Bía Quality Assurance Scheme in Ireland, and the IKB Nederland Varkens in The Netherlands.

Despite these efforts, there is no evidence in the scientific literature of any significant change in swine *Salmonella* infection reduction in pigs or in human cases related to pork consumption, and the overall *Salmonella* seroprevalence remains stable in pigs in many of these non-Scandinavian countries (Correia-Gomes et al., 2021). Only Germany recently reported some positive results after more than 20 years since the implementation of its program (Anonymous, 2021). Meanwhile, the United Kingdom suspended its serological monitoring in 2012 (Anonymous, 2012), and Belgium, which also suspended its serological monitoring, only has maintained veterinary advice on the control of pig salmonellosis (Anonymous, 2015).

The overall lack of efficacy and the high cost of the on-farm control of pig salmonellosis, especially for countries with large pig census (Anonymous, 2011; Gavin et al., 2018), suggest the need to revisit these NCPs. Since in the EU, pig salmonellosis is by far a public health problem, not a pig health problem, a change in the programs' main objective would be advisable. Asymptomatic *Salmonella*-infected pigs commonly arrive at the abattoir for slaughter [European Food Safety Authority (EFSA), 2011], and they are particularly prone to *Salmonella* shedding (Rostagno et al., 2010). Thus, live pigs, through their feces, are a major source of abattoir environmental *Salmonella* contamination, likely being the main source of carcass contamination and, consequently, pork and related products (Argüello et al., 2013a; Swart et al., 2016; Marin et al., 2020). Thus, the main objective of a NCP aimed at reducing the incidence of human salmonellosis may be to focus on finding ways to minimize *Salmonella* contamination in abattoirs, which in the short term, could be more cost-effective than trying to stop the infection within pig farms. Being able to predict the likelihood that a pig will shed *Salmonella* upon its arrival at the abattoir may be the first step to reaching this objective.

Casanova-Higes et al. (2017) observed that pigs shedding *Salmonella* at slaughter seroconverted earlier during the fattening period than non-shedder pigs. A subsequent study showed that on-farm serology could, to some extent, help predict the probability of a pig shedding *Salmonella* at slaughter, thus allowing for the prompt implementation of on-farm and slaughter interventions to reduce the likelihood of abattoir environmental contamination with *Salmonella* (Mainar-Jaime et al., 2018). However, since these studies were carried out on a small number of pig batches from a single *Salmonella*-positive farm and with no additional information, their results should be confirmed further.

Thus, the main objective of this study was to assess whether serology and other farm and/or environmental characteristics (*Salmonella* pen contamination, farm biosecurity, season, etc.) could be used as predictors of *Salmonella* shedding at the abattoir. By predicting the risk of *Salmonella* shedding for a given batch of pigs upon their arrival at the abattoir, subsequent carcass contamination could be prevented by implementing both on-farm and abattoir control strategies.

## 2. Materials and methods

### 2.1. Animal selection and sampling

Between December 2019 and March 2022, 30 batches of 50 pigs each (a total of 1,500 pigs) from 30 different fattening units (average size ≈1,000 pigs/unit) were chosen for this study. Farms were selected based on farmers' willingness to collaborate and the availability of veterinary services.

The 50 animals from each fattening unit were selected approximately 3-4 weeks before slaughter, as suggested in a previous study (Mainar-Jaime et al., 2018). They were chosen from different pens along the fattening units (from 1 to 3 pigs/pen) among the first pigs of the unit to be sent for slaughter (the heaviest ones). At that moment, pigs were ear-tagged, and their blood samples were taken for the detection of specific antibodies against *Salmonella*. In addition, pooled floor fecal (FF) samples were collected from 10 pens distributed at different points of the fattening unit (corners, middle areas, and right and left to aisles) for the detection of *Salmonella* on the farm.

The selected pigs were loaded onto a clean and disinfected truck along with other pigs from the same fattening unit (up to approximately 200 pigs/truck); thus, they were not mixed with pigs from other different units. Transport to slaughter usually occurred on Monday mornings, but they were not necessarily the first batches to be slaughtered that day. In general, farms were not more than 2 h away from the abattoir (mean distance farm to abattoir: 33 km; 95%CI = 25.4–40.5). At the abattoir, pigs were kept in a clean pen without mixing them with pigs of other origins. Slaughtering was performed within the first two hours after arrival. All the procedures followed the usual abattoir routine, and no specific changes were made for this study. Tagged animals were identified at the slaughter line, and after evisceration, a minimum of 25 g of intestinal (colon) content (IC) was collected from the gastrointestinal package of each of these pigs for assessing their *Salmonella* shedding status. After collection, all samples were transported directly to the laboratory for immediate processing.

### 2.2. Farm biosecurity questionnaire

A questionnaire on the different aspects of farm biosecurity was filled in by the veterinarian responsible for each farm included in the study. This questionnaire was based on that available through Biocheck. Gent BV, Belgium (https://biocheckgent.com/en). Briefly, it consisted of a risk-based scoring system and retrieved information on external and internal farm biosecurity. Regarding external biosecurity, the factors considered were the purchase of animals, transport of animals, removal of manure and dead animals, feed, water and equipment supply, personnel and visitors, vermin and bird control, and environmental region. Regarding internal biosecurity, the factors considered were disease management, fattening unit management, measures between compartments and the use of equipment, and cleaning and disinfection. For each category, a score between 0 for the worst scenario and 100 for the best biosecurity level was obtained. A final score on the overall farm biosecurity level, which was computed as the average of external and internal biosecurity scores, could then be calculated. These results could be further compared to national score averages.

### 2.3. Serological analysis

Sera were analyzed by an indirect enzyme-linked immunosorbent assay (ELISA) for the presence of specific antibodies against *Salmonella* (Herdcheck Swine *Salmonella* test, IDEXX Laboratories, Westbrook, ME, USA). This test is designed to detect antibodies to the LPS *Salmonella* B, C1, and D serogroups (O-antigens 1, 4, 5, 6, 7, and 12), which are the most common serotypes isolated in pigs. Individual results were presented as optical density percentages (OD%) as compared to the control sera. For farm seroprevalence estimates, a high cut-off value (OD% ≥40) was used to deem a pig as seropositive. Given the limited test's sensitivity and specificity on field samples (73% and 95%, respectively; Mainar-Jaime et al., 2008), high OD% values allowed for the minimization of the number of false-positive individual results.

### 2.4. *Salmonella* isolation and identification of the main serotypes

*Salmonella* identification from FF and IC samples was carried out following the standard ISO 6579-1:2017 method. A colony from each *Salmonella*-positive culture was selected for PCR identification of the two major serotypes of concern in the pig industry, i.e., *S*. Typhimurium and the mST. These two serotypes are the second and third most prevalent in human cases, and a high proportion of them are related to pig sources (EFSA and ECDC, 2022). For that purpose, a duplex PCR that simultaneously amplifies a fragment between the genes *fljB* and *fljA* and the phase-2 flagellar gene (*fljB*) was used (Tennant et al., 2010; Barco et al., 2011).

### 2.5. Pulsed-field gel electrophoresis

Cross-contamination may occur during transport and lairage (Argüello et al., 2013a). Therefore, to confirm the spread of isolates from the farm to the slaughter, the genetic relationship between the *Salmonella* strains shed by pigs at slaughter (IC samples) and those isolated from farm FF samples was assessed by performing PFGE analysis (Ribot et al., 2006). PFGE analysis was performed on isolates identified as *S*. Typhimurium or mST.

Only isolates from FF samples and IC samples from the same farm that showed the same serotype were analyzed. If several isolates met this criterion, then a maximum of three pig isolates and three FF isolates per farm were analyzed. PFGE pattern analysis was performed using the BIONUMERICS software (version 6; Applied Maths, Sint-Martens-Latem, Belgium) with Dice's coefficient and the unweighted pair group method with arithmetic averages (UPGMA dendrogram type), employing a position tolerance of 2.0% and optimization of 2.0%. Fragments less than 30 kb long were not included in the final analysis as they are produced by plasmid DNA (Kariuki et al., 2000; Peters et al., 2003; Cooke et al., 2008).

### 2.6. Statistical analyses and *Salmonella* shedding predictive model development

Estimates of on-farm *Salmonella* seroprevalence, pen prevalence, and prevalence of *Salmonella* shedding at slaughter with their corresponding 95% confidence intervals (95%CI) were calculated for the fattening units.

To create the predictive model for *Salmonella* shedding at slaughter, a random-effects logistic regression analysis was conducted, with *Salmonella*-shedding pigs at the abattoir (yes/no) as the dependent variable. Serology (OD% values), internal, external, and total biosecurity, percentage of *Salmonella*-positive pens in the farm (three categories: no positive pens, less than 20% of positive pens, ≥30% of positive pens), and other farm and/or environmental characteristics that may be related to *Salmonella* infection, such as the season of sampling (spring, summer, autumn, and winter), the distance (in km) and time (in minutes) of transport from the farm to the abattoir, and the time elapsed between farm and abattoir samplings (in days) were the independent variables. The presence of *S*. Typhimurium/mST in the farm (yes/no) was also included in the model as *Salmonella* shedding, and immune response could be related to these serotypes (Ivanek et al., 2012). Since animals were grouped within farms, the farm was considered a random (grouping) variable to account for the correlation between individual pigs coming from the same farm (i.e., intraclass correlation, ICC). Two-thirds of the study population, which was defined as the total number of pigs for which all required information was obtained, were randomly selected to run the main model. The remaining third of the pigs were used for model validation (reproducibility).

Receiver operating characteristic (ROC) curves were built from potential logistic models, and the area under the curve (AUC) was used as a method for selecting the best predictive model (Greiner et al., 2000). Final estimates of the probability of shedding *Salmonella* were calculated for each pig from the selected logistic regression equation, and different scenarios were identified according to the different levels of the variables included in the model. Furthermore, a cut-off value was selected, based on Pythagoras' theorem-based method, which is a new approach based on the smallest sum of squares of 1-sensitivity and 1-specificity, to assess the diagnostic accuracy of the prediction when sensitivity (Se) and specificity (Sp) were valued equally. The cut-point chosen by this method always selects the one closest to the top-left corner of the ROC curve, regardless of its shape (Froud and Abel, 2014).

Statistical analyses were performed using the STATA software (STATA/IC 12.1. StataCorp. LP, College Station, TX, USA) and MedCalc^®^ statistical software version 20.215 (MedCalc Software Ltd, Ostend, Belgium).

## 3. Results

### 3.1. Farm biosecurity and *Salmonella* contamination at the farm

**Figure 1:**
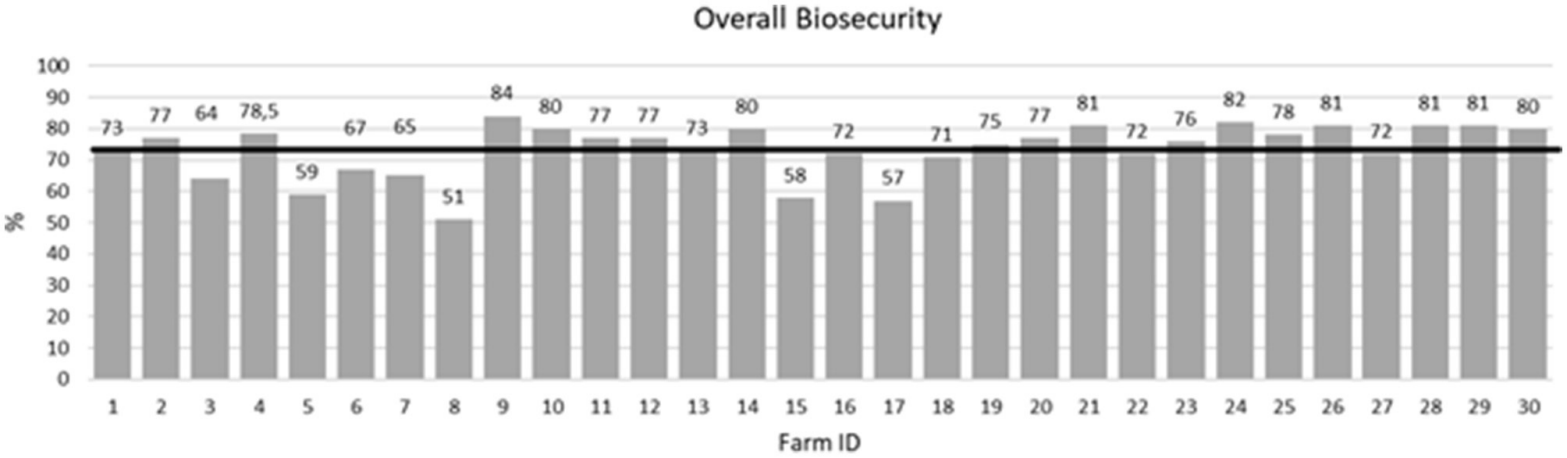
Individual overall biosecurity scores for the 30 pig farms included in the study and the mean value (solid black line) of all farms.

The 30 selected farms presented a mean overall biosecurity score of 73.31% (ranging from 51% to 84%) (Figure 1). The mean internal biosecurity score for these farms was 67.80% (min.: 40%, max.: 82%), while their mean external biosecurity score was 78.98% (min.: 62%, max.: 89%).

*Salmonella* was detected in 13 (43.3%; 95%CI = 27.4–60.8) of the fattening units sampled, with a mean pen prevalence of 36.9% (95%CI = 22.6–51.2) in the *Salmonella-*positive units. *S*. Typhimurium was present in six (46.2%), the mST in eight (61.5%), and other serotypes in only two (15.4%) of the pig units. Seven (53.8%) of the *Salmonella*-positive units showed ≥30% of positive pens.

*Salmonella* was recovered from 48 out of 300 pooled FF samples (16%; 95%CI = 12.3–20.6) analyzed. Among the positive FF samples, *S*. Typhimurium was isolated in 21 samples (43.7%; 95%CI = 30.7–57.7), and the mST was isolated in 20 samples (41.7%, 95%CI = 28.9–55.7). *Salmonella* isolates belonging to serotypes other than these two were found in only seven FF samples (14.6%; 95%CI = 7.3–27.2).

### 3.2. *Salmonella* farm seroprevalence

**Figure 2:**
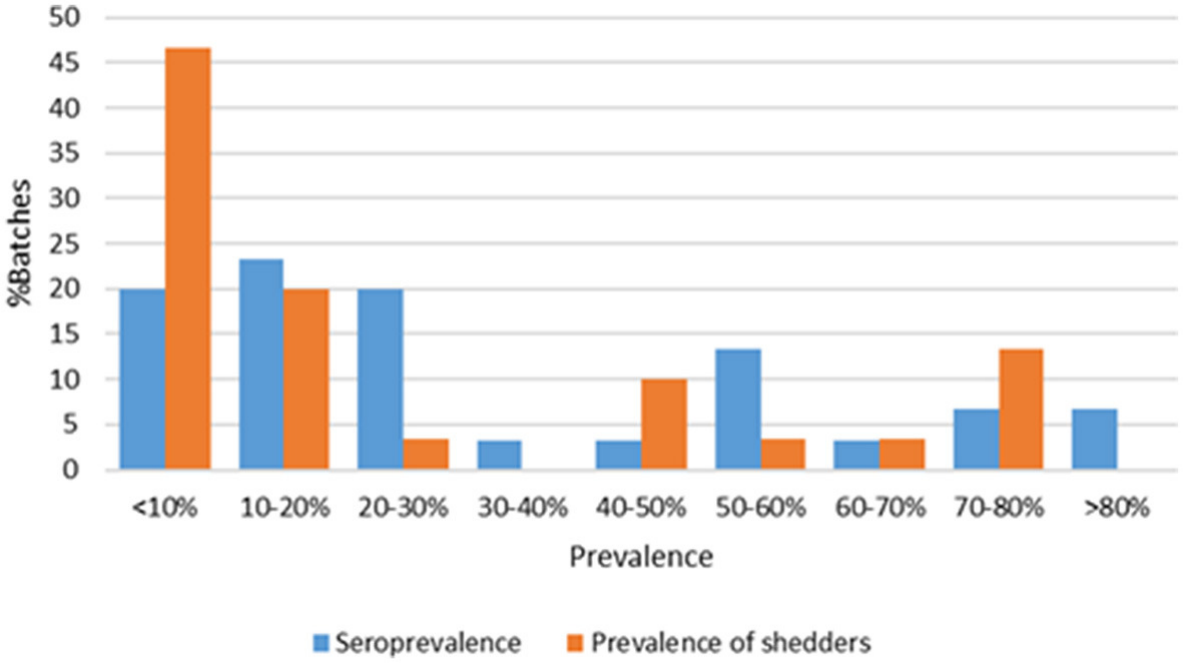
Distribution of *Salmonella* seroprevalence and the prevalence of *Salmonella* shedders among the 30 pig batches. Spearman's coefficient of rank correlation (rho) between on-farm seroprevalence and the prevalence of *Salmonella* shedders at slaughter: 0.451; 95%CI = 0.11–0.69; *P* = 0.012.

Out of the 1,500 pigs initially ear-tagged at the farm, a total of 1,341 (89.4%) were further identified at slaughter, and IC samples were collected (an average of 44.7 pigs/fattening unit; 95%CI = 42.6–46.8). Serological analyses were performed on these 1,341 pigs. The mean seroprevalence (cut-off value OD% ≥40) among the 30 units was 31.7% (95%CI = 21.8–41.0), but it differed significantly among farms, ranging from a minimum of 2.3% to a maximum of 87.0%. Only six of these farms showed seroprevalences below 10%. The distribution of the seroprevalence among pig farms is shown in Figure 2.

### 3.3. Prevalence of *Salmonella* shedding at the slaughterhouse

A total of 316 pigs (23.6%; 95%CI = 21.4–25.9) were shedding *Salmonella* at slaughter. The prevalence of shedding differed significantly among farm batches, ranging from 0% to a maximum of 79.4%. All pigs were *Salmonella* negative only in three of these batches. The distribution of *Salmonella* shedding prevalence among pig batches is shown in Figure 2.

The major serotype identified was the mST, which was isolated in 151 pigs (47.8%; 95%CI = 42.3–53.3). *S*. Typhimurium was isolated in 71 pigs (22.5%; 95%CI = 18.2–27.4). Serotypes other than *S*. Typhimurium and the mST were identified in 94 pigs (29.7%; 95%CI = 25.0–35.0).

### 3.4. PFGE

Since *S*. Typhimurium and the mST were the two major serotypes involved (isolated in >80% PFF samples and 70% IC samples), PFGE analysis was performed on these *Salmonella* serotypes but only when the same *Salmonella* serotype was detected in both FF and IC samples from the same fattening unit. A total of 20 *Salmonella* isolates from FF samples and 26 isolates from IC samples from nine *Salmonella*-positive units were submitted for PFGE analysis.

**Figure 3:**
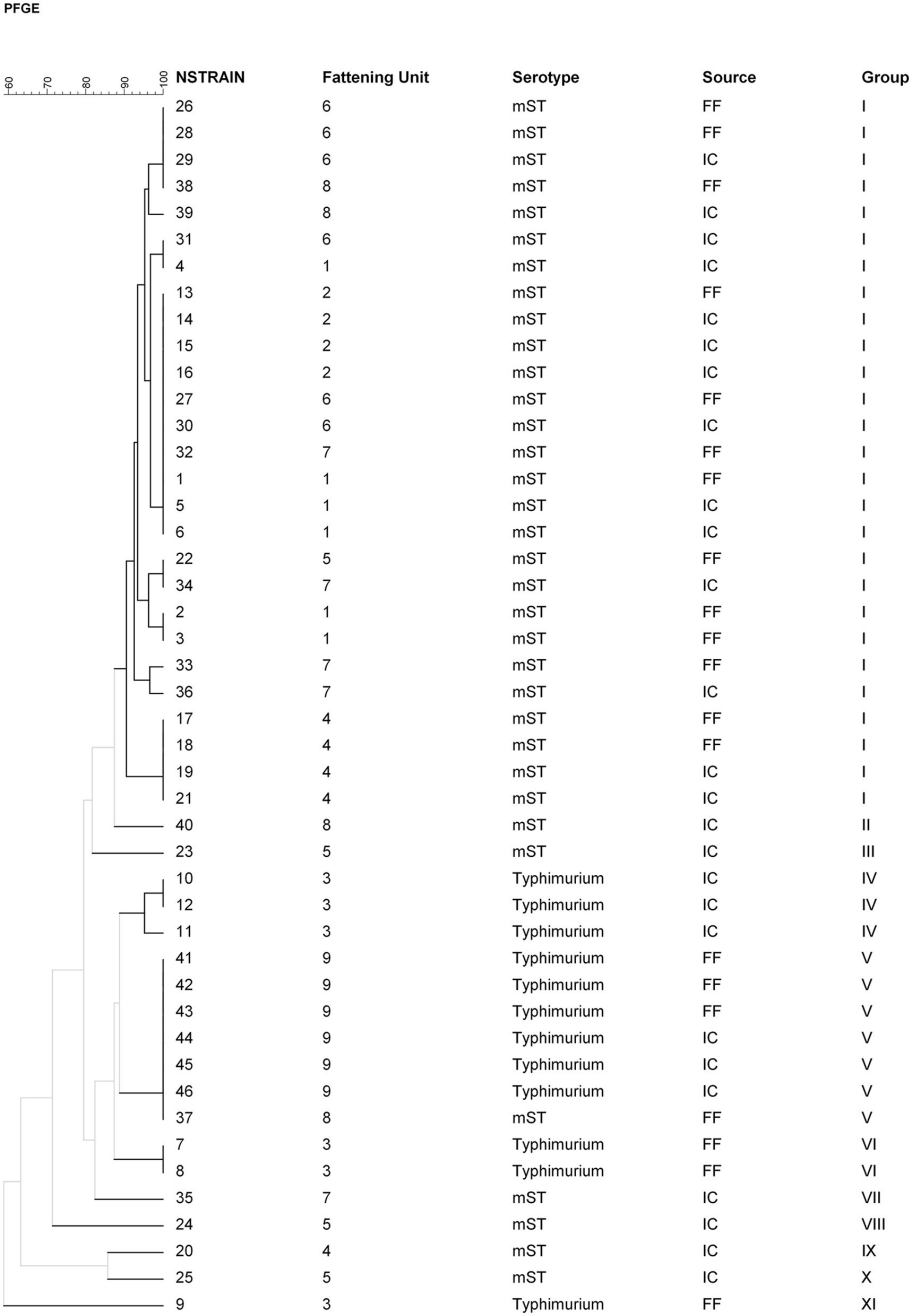
Dendrogram showing the main *XbaI* pulsed-field gel electrophoresis (PFGE) patterns (≥90% homology) for 46 *Salmonella* strains isolated from slaughtered pigs' intestinal content (IC) and pen floor fecal (FF) samples from 9 fattening units.

PFGE analysis showed 11 different *XbaI* patterns (based on a similarity cut-off of ≥90%) (Figure 3). The observed PFGE clusters matched well with the serotypes. Four main clusters were observed for *S*. Typhimurium (clusters IV, V, VI, and XI), including only three farms, and seven for mST (clusters I, II, III, VII, VIII, IX, and X).

*Salmonella* isolates from FF samples were grouped into four different PFGE patterns (I, V, VI, and XI). Clusters VI and XI were composed only of isolates from FF samples. Within the other two clusters (clusters I and V), isolates from IC samples obtained from pigs from the corresponding fattening unit were also included. Overall, 65.4% of the IC isolates analyzed were included within these two clusters. At least one genetic relationship between *Salmonella* isolates from IC and FF samples was detected in 77.7% of the pig units.

### 3.5. *Salmonella* shedding prediction model development and validation

Two-thirds (885 animals) of the 1,341 pigs were randomly selected for building the predictive model. The results of the random-effects logistic regression analysis showed three variables related to *Salmonella* shedding at the abattoir, namely, serology (included as the logarithm of OD% values), the percentage of *Salmonella*-positive pens in the farm (used as a categorical variable based on percentiles: no positive pens; low percentage of positive pens, ≤ 20%; and high percentage of positive pens, >20%), and the internal biosecurity score (also used as a categorical variable based on percentiles: low score, < 64%; medium score, from 64 to 77%; and high score, >77%) (Table 1). The random-effects logistic regression analysis indicated a significant clustering effect of “Farm” (ICC = 0.33; *P* < 0.001).

**Table 1:** Results of the random-effects logistic regression analysis^*^ for predicting *Salmonella* shedding at the abattoir.

	**Odds ratio (OR)**	***P*-value**	**95% CI(OR)**
Serology (LogOD%)	1.74	0.028	1.06–2.87
**%** ***Salmonella*****-positive pens**			
0^a^	1	-	-
10–20	5.46	0.029	1.19–24.95
≥30	8.18	0.003	2.07–32.33
**Internal biosecurity score**			
< 64%^a^	1	-	-
64–77%	0.25	0.043	0.07–0.96
>77%	0.20	0.050	0.04–0.99
Constant	0.11	0.005	0.03–0.52

^*^Farm regarded as a grouping (random) variable.

^a^Reference category. Intraclass correlation coefficient (ICC): 0.33 (95%CI = 0.19–0.50).

Individual serology was positively related to *Salmonella* shedding at the abattoir as increasing OD% values increased the odds of shedding (OR = 1.75; 95%CI = 1.06-2.87). Pigs from fattening units in which *Salmonella* was isolated from 10 to 20% of the pens had approximately five times higher odds of shedding *Salmonella* at the abattoir than pigs from units where *Salmonella* was not isolated from any pen (OR = 5.46; 95%CI = 1.19–24.95). These odds were even higher when *Salmonella* was detected in ≥30% of the pens in the unit (OR = 8.18; 95%CI = 2.07–32.33; *P* < 0.01). Regarding farm biosecurity, medium and high internal biosecurity scores significantly decreased the odds of a pig shedding *Salmonella* when compared to low biosecurity scores (OR = 0.25; 95%CI = 0.07–0.96 and OR = 0.20; 95%CI = 0.04–0.99, respectively).

**Figure 4:**
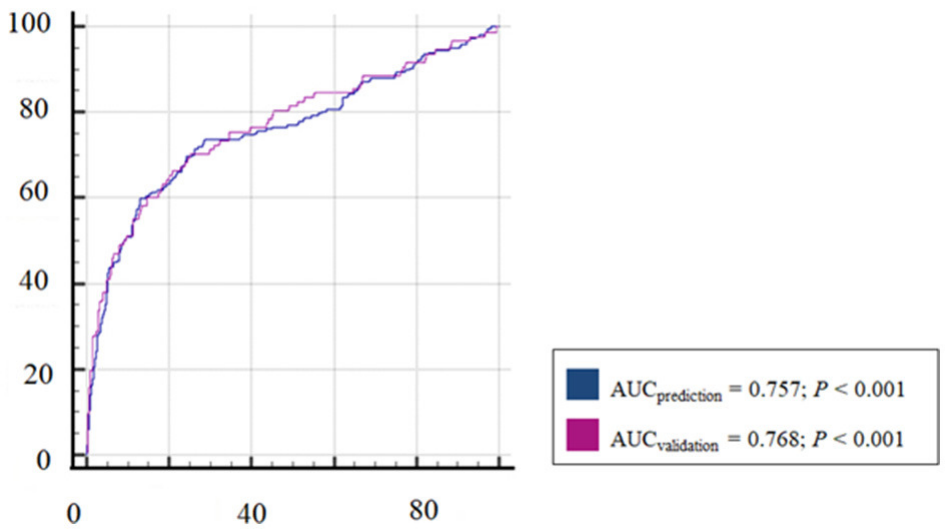
Receiver operating characteristic curves (ROC) and the corresponding areas under the curve (AUC) for the estimated (blue) model and the validation model (purple).

The prediction model built with these three factors showed a good ability to predict whether an animal will shed *Salmonella* at the abattoir. The AUC (0.76) was significantly different from that for a non-discriminatory model (Figure 4). The best cut-off value for maximizing Se (i.e., its ability to correctly identify an animal that will be shedding *Salmonella* after its arrival to the abattoir) and Sp (i.e., its ability to correctly identify an animal that will not shed *Salmonella* at the abattoir) was 25.9%. The associated diagnostic Se and Sp for that cut-off value were 71.6% and 73.6%, respectively (Table 2).

**Table 2:** Prediction parameters for the estimated model and the validation model.

	**Estimated model**	**Validation model**
AUC	0.757	0.768
95% CI (AUC)	0.728–0.785	0.727–0.806
Pythagoras cut-off^*^	25.88	26.47
Sensitivity	71.56	70.41
95% CI (sensitivity)	65.1–77.4	60.3–79.2
Specificity	73.61	74.58
95% CI (specificity)	70.1–76.9	69.7–79.0

AUC, Area under the curve.

^*^Estimated as the smallest sum of squares of 1-sensitivity and 1-specificity (Froud and Abel, 2014).

The model was rerun using data from the one-third left of the pig population (*N* = 456) for validation purposes. The comparison between the predictive and the validation models showed non-significant differences (Table 2 and Figure 4).

**Figure 5:**
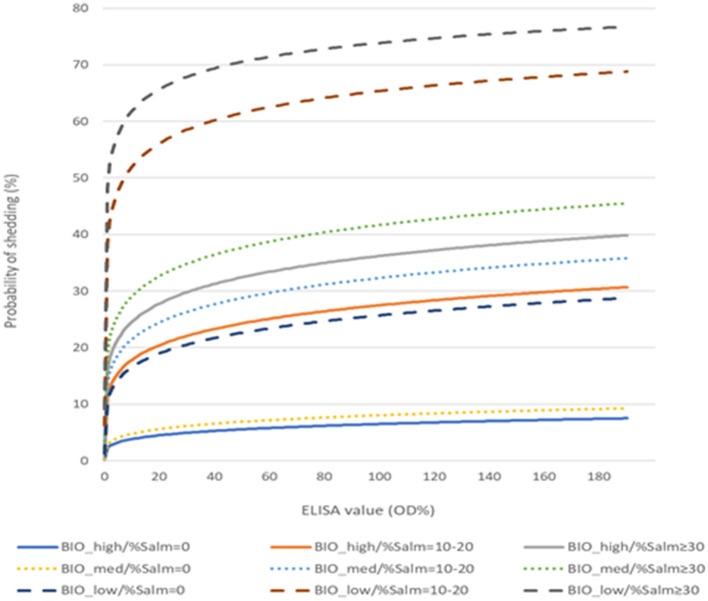
Probability of *Salmonella* shedding at the abattoir as a function of serology (ELISA OD% values), farm internal biosecurity (BIO: high, medium, and low), and the percentage of *Salmonella*-positive pens in the farm (%Salm: 0%, 10–20% and ≥30%).

Figure 5 shows the predicted probability of shedding *Salmonella* at the abattoir according to serological values (ELISA OD%) after considering the proportion of *Salmonella-*positive pens in the fattening units and their internal biosecurity scores. In two scenarios, serology would not add significant information for considering whether a pig would shed *Salmonella* at the abattoir: in the case of farms with high or medium internal biosecurity with no *Salmonella*-positive pens or in the case of farms with low internal biosecurity with at least one *Salmonella*-positive pen.

## 4. Discussion

In this study, a total of 1,341 pigs from 30 farms that were willing to participate and were located in Northeast Spain, the largest pig production region in Spain (MAGRAMA, 2021), were selected. Even though pig salmonellosis is considered a public health concern, *Salmonella* contamination was detected in 43.3% of the farms, a figure comparable to that reported in 2003-2004 in a similar study on the entire country (García-Feliz et al., 2007). More concerning, the proportion of pigs shedding *Salmonella* at the abattoir was also high (23.6%), with some pig batches reaching up to 80% and with zoonotic *S*. Typhimurium and the mST being the predominant serotypes. These pigs are likely major sources of carcass contamination (Argüello et al., 2013a; Marin et al., 2020), and finding ways to prevent this shedding should be of utmost importance.

As indicated by the random-effects logistic model, the proportion of pigs shedding *Salmonella* at the abattoir was strongly related to the presence of *Salmonella* in the fattening unit from which pigs came (Table 1). This relationship was supported to some extent by the identification through PFGE analyses of genetic matches between *Salmonella* isolates from pen FF and pig IC samples in most of the analyzed batches (Figure 3), despite the possibility of cross-contamination during transport or lairage (Argüello et al., 2013a). These findings emphasized the role that *Salmonella* farm contamination plays in *Salmonella* shedding at slaughter and suggested that *Salmonella* control should begin at the farm (de Busser et al., 2013).

A thorough biosecurity questionnaire was carried out on all the pig farms to describe their overall biosecurity level. On average, they presented good biosecurity levels. The mean biosecurity score for these farms was 73%, which appears to be somewhat higher than the Spanish national average (68%, from 275 questionnaires) and even higher than the national average for other big European pig-producer countries such as Germany (64%; 180 questionnaires), The Netherlands (69%; 198 questionnaires), Italy (71%; 353 questionnaires), or Ireland (72%; 486 questionnaires). However, the score was lower than that in Belgium (75%; 10,068 questionnaires) (as checked at biocheck.ugent.be on 31 January 2023). However, despite this good level of overall biosecurity, serological results suggested that *Salmonella* was circulating within most farms, highlighting the difficulties in controlling it at the farm level. On average, 32.9% of the pigs presented high ELISA OD% values (≥40%). Based on these results, one-third of the farms would be classified within the high-seroprevalence category (≥40% seroprevalence), and another one-third would be classified within the medium category (between 20% and 40% seroprevalence), according to the main NCPs. Only six farms showed seroprevalence levels below 10%. These results might also explain to some extent the high proportion of pigs shedding *Salmonella* at the abattoir, as a significant positive but weak association was observed in the logistic model between pig serology and *Salmonella* shedding at slaughter (Table 1). Pigs with much higher ELISA OD% values would have somewhat higher odds of shedding *Salmonella* at slaughter (Kranker et al., 2003; Sørensen et al., 2004; Korsak et al., 2006; Mainar-Jaime et al., 2018).

In this study, neither the overall nor the external biosecurity scores were related to the reduction of *Salmonella* shedding at the abattoir (Table 1). Although the biosecurity level of farms is, in general, considered to be beneficial to reducing bacterial transmission, it appears that biosecurity cannot reduce *Salmonella* prevalence by itself (Alarcón et al., 2021; Youssef et al., 2021). The implementation of efficient on-farm *Salmonella* control measures will depend on farmers' perception of the disease and their motivation to maintain them on an ongoing basis (Fraser et al., 2010; Marier et al., 2016). However, pig salmonellosis is usually asymptomatic, that is, of low concern for farmers and swine production veterinarians.

The predictive model built with these three factors, i.e., serology, farm internal biosecurity, and *Salmonella* pen prevalence, showed an acceptable ability to predict whether an animal will shed *Salmonella* at the abattoir. Thus, according to the model, a pig with a predicted probability of shedding *Salmonella* greater than 26% would have a 71.6% probability of being a true shedder. If the predicted probability was ≤ 26%, the animal would have a 73.6% probability of being a true non-shedder. Therefore, estimating the proportion of animals with a model probability higher than 26% in a given batch of pigs intended for slaughter would allow for the assessment of the overall risk of shedding for that batch. Once the potential risk of *Salmonella* shedding has been assessed 3-4 weeks before slaughter, stakeholders could act according to the results obtained. At the farm level, control measures could be implemented to minimize the likelihood of *Salmonella* shedding at slaughter. For instance, the addition of organic acids in food/water could help reduce shedding (de Busser et al., 2009; Argüello et al., 2013b; Lynch et al., 2017; Bernad-Roche et al., results to be published). Additionally, the abattoir, being aware of the risk, could implement mitigation measures such as logistic slaughter (Swanenburg et al., 2001; Hotes et al., 2011) or even the addition of organic acids to the water at lairage (Bernad-Roche et al., 2022), to attempt to reduce the risk of *Salmonella* shedding. These interventions will only be required on those batches of pigs that present probabilities of *Salmonella* shedding above an established threshold.

It is interesting to note that in farms with high or medium internal biosecurity scores (i.e., scores >64%) and where no *Salmonella*-positive pens were detected, no animals could be considered shedders at slaughter (i.e., with predicted probability >26%), regardless of the ELISA OD% values (Figure 5). Likewise, serology would be unnecessary in the case of farms with low internal biosecurity when at least one pen is positive for *Salmonella*, as all pigs would show high predicted probabilities of shedding the bacterium at slaughter. In this second scenario, the likelihood of a pig becoming infected will be high, since *Salmonella* should be able to circulate easily among pens in farms with low internal biosecurity (Baptista et al., 2010).

These results emphasized the importance of internal biosecurity in the transmission of *Salmonella* within the farm. The internal biosecurity questionnaire included factors such as disease management (i.e., vaccination and treatment protocols and frequency of health status assessment), fattening unit management (i.e., all-in/all-out system and pig mix and density), measures between compartments and the use of equipment (i.e., foot baths, cleaning and disinfection after equipment usage, workflow from younger to older pigs, and sharing equipment with other farms), and cleaning and disinfection (i.e., after every production cycle, protocols, and drying after cleaning and disinfection). Cleaning and disinfection along with feed, water, and bedding are considered by experts some of the most important biosecurity measures to control *Salmonella* in indoor settings (De Lucia and Ostanello, 2020; Galipó et al., 2023). However, more research is required to determine with more certainty the most-effective measures from the human health perspective (Youssef et al., 2021).

In contrast to the two previous scenarios, in the case of any of the other situations (i.e., acceptable internal biosecurity with the presence of *Salmonella*-positive pens or low internal biosecurity and no *Salmonella*-positive pens), serology would add useful information to the decision of whether a pig should be considered of risk for *Salmonella* shedding at the abattoir. As an example, pigs with ELISA values >30% would be associated with abattoir *Salmonella* shedding if they belong to farms with medium internal biosecurity, along with the presence of *Salmonella* in 10–20% of the pens. If they belong to a similar farm but with high internal biosecurity, the pigs of risk would be those showing much higher ELISA values (close to 80%). ELISA values would also help interpret the possible shedding status of a pig from a low internal biosecurity farm when no *Salmonella*-positive pens are detected (Figure 5). Therefore, considering the different scenarios that are possible according to the model, the simplest way to proceed with the estimation of the risk of *Salmonella* shedding at slaughter would be to start with an assessment of the farms' internal biosecurity and the *Salmonella* pen prevalence. Depending on the results, additional serological analyses should be carried out on a representative sample of the pigs from the batch.

Although many studies have shown the lack of reliability of indirect ELISA tests for ascertaining the individual *Salmonella* status of a pig, either infection or shedding (Nollet et al., 2005; Farzan et al., 2007; Gradassi et al., 2015), they could be of relative value when used on groups of pigs (Sørensen et al., 2004; Korsak et al., 2006; Farzan et al., 2007). In this study, a significant but low correlation was observed between the *Salmonella* seroprevalence of a given batch and the proportion of shedding pigs at the abattoir for that batch (Figure 2), when no other factors were taken into account. This simple approach might not be sufficient for accurately predicting *Salmonella* shedding at the abattoir.

It appears that the value of serology for predicting shedding depends on the context in which it is used. Similar to the results of a previous study (Mainar-Jaime et al., 2018), the model in this study indicated that higher individual OD% values were related to higher odds of *Salmonella* shedding at slaughter. This was likely due to the presence of stressful factors such as the transport and waiting (lairage) times that pigs went through, which would favor the shedding among the infected pigs (Duggan et al., 2010; Simons et al., 2016). In addition, other factors, such as the internal biosecurity of the farm and the presence of *Salmonella* in the farm, would also play a significant role in interpreting ELISA values in this context. Thus, these results might help explain, at least in part, the difficulties that many NCPs face in properly assessing the *Salmonella* status of pig farms, as many of them are based exclusively on serological results usually obtained from a small and hardly representative number of animals, without considering other factors (Mainar-Jaime et al., 2018; Correia-Gomes et al., 2021).

## 5. Conclusion

This study highlighted the importance of the context in which serology is used for pig salmonellosis control. Using it 3-4 weeks prior to sending the pigs to slaughter might be helpful for identifying batches of pigs at risk of shedding *Salmonella* upon their arrival at the abattoir. In some cases, being aware of the farm's internal biosecurity level and performing a bacteriological sampling of a representative number of pens might be sufficient for estimating the risk of *Salmonella* shedding for a given batch of pigs ready for slaughter. In others, serology would be required for a more accurate interpretation of the results, but in both situations, an acceptable level of knowledge about the risk of *Salmonella* shedding for a given batch of slaughter pigs could be achieved. Reducing the likelihood of *Salmonella* shedding at this stage would be an important step for reducing *Salmonella* carcass contamination.

An additional advantage of this approach is that *Salmonella* control would not initially rely on the farmer's work but on the farm's data collection, as farmer's engagement seems to be one of the main obstacles NCPs face, especially when dealing with animal infections of no clinical concern (Fraser et al., 2010; Marier et al., 2016; Alarcón et al., 2021). Moreover, this approach would also allow for a combined farm/abattoir strategy that would likely have cumulative benefits (Swart et al., 2016), as it would make both abattoirs and farmers aware of the risk of the pigs coming to the slaughter. Thus, a more precise characterization of the *Salmonella* status of pig farms would be obtained from routine sampling, with the collection of proper representative samples, which would also help encourage a good attitude among farmers toward *Salmonella* control in the short/medium term.

## Data availability statement

The raw data supporting the conclusions of this article will be made available by the authors, without undue reservation.

## Ethics statement

The animal study was approved by Ethical Advisory Commission for Animal Experimentation of the University of Zaragoza (permit no. PI13/20). The study was conducted in accordance with the local legislation and institutional requirements.

## Author contributions

RM-J: conceptualization, supervision, project administration, and funding acquisition. RM-J and MB-R: methodology, investigation, and writing-original draft preparation. RM-J, MB-R, IB, and AC-S: formal analysis. MB-R, RM-J, and CM-A: writing-review and editing. All authors have read and agreed to the published version of the manuscript.
